# G‐space versus E‐space: Are hornets (Hymenoptera: Vespidae) at equilibrium with honeybees in Asia?

**DOI:** 10.1002/ece3.11615

**Published:** 2024-06-30

**Authors:** Ehsan Rahimi, Chuleui Jung

**Affiliations:** ^1^ Agricultural Science and Technology Institute, Andong National University Andong Korea; ^2^ Department of Plant Medical Andong National University Andong Korea

**Keywords:** Asia, E‐space, G‐space, honeybees, hornets, niche conservatism, niche overlap

## Abstract

This study delves into the concept of niche conservatism (NC) and its implications for how hornets (Hymenoptera: Vespidae) and honeybees respond to climate change. Our primary objectives are threefold: firstly, to assess whether distinct environmental niche spaces (E‐space) exist between 12 hornets and honeybees like *Apis cerana* and *Apis mellifera* in Asia; Secondly, to explore the degree to which Asian hornets have attained geographic equilibrium alongside honeybee species. Lastly, is to investigate how the geographic niche overlap (G‐space) between hornets and honeybees could potentially change under climate change scenarios. To accomplish these goals, we employed ordination and ecological niche modeling techniques to analyze 91 pairs of hornets and honeybees in both geographic (G‐space) and environmental (E‐space) contexts. Then, we projected the potential impacts of climate change on the future geographic overlap between hornets and honeybees, specifically under the SSP585 climate scenario for the year 2070. Our results demonstrated that the environmental niches (E‐space) of hornets and honeybees can be treated as interchangeable, indicating they have similar environmental preferences despite being unrelated taxa. We found that *Vespa velutina* currently exhibits a moderate geographic niche overlap (G‐space) of 0.63 with both honeybee species. Meanwhile, *Vespa mandarinia* demonstrates an overlap of 0.46 with *Apis cerana* and 0.63 with *Apis mellifera*. The overlap of *Vespa velutina* with *Apis cerana* might potentially decrease to 0.51 and 0.56 with *Apis mellifera*. For *Vespa mandarinia*, the overlap could reach 0.41 with *Apis cerana* and 0.6 with *Apis mellifera* under a climate change scenario. This study indicates that the limited spatial overlap between honeybees and hornets across certain areas in Asia is more likely influenced by geographical barriers rather than solely environmental unsuitability for hornets. In this study, we delve into the concept of niche conservatism (NC) and its implications for how hornets (Hymenoptera: Vespidae) and honeybees respond to climate change.

## Graphical abstract

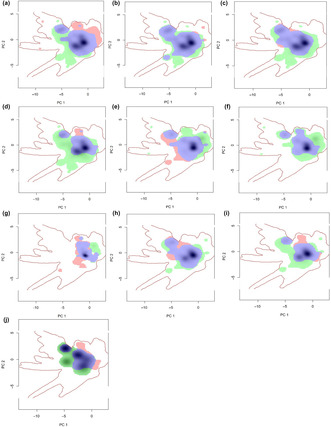

## 1 INTRODUCTION

The tendency of species to retain aspects of their fundamental niche over time is called niche conservatism (NC) (Wiens & Graham, 2005). If fundamental niches of species are conserved, species will only be able to invade regions that have a climate similar to that of their native range (Tirozzi et al., 2022). Moreover, if climatic tolerances are conserved, species should shift their geographic ranges in the face of global warming to track their ancestral climatic regime. Specifically, they would be expected to move poleward in latitude and upward in elevation (Rahimi & Jung, 2024); species that are unable to adapt or cannot shift their geographic ranges (e.g., due to habitat destruction or geographic constraints) may be at risk of extinction (Hellmann et al., 2008). Similarly, if NC in climatic tolerance determines the range limits of species, then we should expect to see consistent parallels between their climatic distribution in their native and introduced ranges (Wiens & Graham, 2005). In other words, if climatic conditions in the invaded region are similar to those of the native range, then the species follows NC. In contrast, significant disparities in climatic conditions between the native and introduced ranges indicate that the ecological niche of the species has diverged in its introduced range (Petitpierre et al., 2012).

If climatic tolerances are conserved, the distribution of species in their native ranges should predict where they can successfully invade and subsequently spread (Bock et al., 2016; Wiens et al., 2010). For biogeographic hypotheses, a key concept is that climatically unsuitable conditions can limit geographic ranges when there is NC, and such conditions can potentially be identified and tested using species distribution models (SDMs) (Strubbe et al., 2013). For example, an assumption of climatic NC predicts that invasive species will spread primarily into regions that are climatically similar to their native range (Wiens et al., 2010). Warren et al. (2008) proposed that the detection of NC entails testing two hypotheses, namely niche equivalency (whether native and non‐native niches are indistinguishable) and niche similarity (whether niches are more similar than expected by chance).

For testing NC, we need to quantify the niche breadth of species and the extent to which their native and introduced niches overlap. Niche breadth measures the range of environmental conditions or resources within the ecological niche that a species can utilize and in which it can survive, with a broad niche breadth indicating the ability of a species to tolerate a wide range of conditions or resources. Niches can be compared in geographic space (G‐space) or environmental space (E‐space). The G‐space concept relates to the comparison of occurrences of several species in the same geographic area. E‐space, on the other hand, involves a comparison of environmental conditions, such as climate, soil type, vegetation, or other ecological factors, in either the same geographic area or between different geographic areas (i.e., native and introduced regions) for a single species. The use of E‐space permits better assessment of niche overlap because it takes into account environmental availability and the analogy between ranges (Tirozzi et al., 2022).

The ecology of invasive species is currently a focal point for researchers, with considerable emphasis on predicting the potential distribution of invasive species using niche overlap (E‐space) analyses. However, the field has somewhat overlooked the crucial dimension of biotic interactions, particularly those involving prey–predator dynamics. By quantifying niche overlap in both geographic (G‐space) and environmental (E‐space) contexts for these interactions, it may be possible to glean valuable insights into the impact of invasive species on ecosystems. For instance, when two predator and prey species share similar climatic habitats (indistinguishable E‐space niches) if a non‐native predator species enters the prey's territory, it may occupy all the areas where the native predator resides. Thus, expanding research efforts to incorporate prey–predator interactions within the niche overlap framework is a promising avenue for advancing our understanding of invasion ecology.

Among invasive species, social wasps (Hymenoptera: Vespidae) are some of the most notorious. They are easily recognizable predators that, in temperate regions, form moderate to large annual eusocial colonies. Several aspects of their biology contribute to their effective colonization of habitats in regions where they are not native. Hornets (genus *Vespa*) are represented by 22 species, all of which inhabit various regions of Asia (Archer & Penney, 2012; Smith‐Pardo et al., 2020). Only two species, *Vespa crabro* and *Vespa orientalis*, naturally occur in Europe and no species are native to the Americas. In recent years, several *Vespa* species have been transported beyond their natural ranges (Otis et al., 2023). At least five of those have successfully established non‐native populations and, in doing so, have the potential to disrupt local ecosystems by impacting biodiversity and interfering with pollination systems (Otis et al., 2023).

These undesirable consequences have been detected in regions invaded by *Vespa velutina*, which has successfully colonized most of western Europe, South Korea, and southern regions of Japan (Choi et al., 2012; Lima et al., 2022; Otis et al., 2023; Rahimi et al., 2022), and has recently been detected in the southeastern USA (Alaniz et al., 2021; Otis et al., 2023; Smith‐Pardo et al., 2020). This invasive hornet species has had a highly detrimental impact on local ecosystems, particularly honeybee colonies in these areas (Laurino et al., 2019; Requier et al., 2019; Ruiz‐Cristi et al., 2020). For example, in Europe, *Vespa velutina* causes losses between 18% and 50% of bee colonies (Lima et al., 2022; Zhu et al., 2020). This phenomenon underscores the potential ecological impacts associated with the expansion of hornet species beyond their original ranges (Otis et al., 2023). *Vespa velutina* is also a frequent and effective hunter of flower visitors, leading to changes in the foraging behavior of various pollinator groups (Rojas‐Nossa et al., 2023; Rojas‐Nossa & Calviño‐Cancela, 2020). In France, Our Rome et al. (2021) revealed that *Vespa velutina* exhibits generalist predation behavior, targeting honeybees (38.1%), flies (29.9%), social wasps (19.7%), and a diverse array of other animal organisms. In Asia, recognized as the epicenter of hornet evolution (Matsuura & Yamane, 1990), native honey bee species like *Apis cerana* or *A. dorsata* have developed effective defenses to protect their colonies against hornet attacks (Cappa et al., 2021). In contrast, European honey bees (*Apis mellifera*), have exhibited comparatively weak defense strategies (Jung & Cho, 2015).

In Europe, Lioy et al. (2023) examined the realized climatic niche of *Vespa velutina nigrithorax* and its response to various climatic conditions, while also assessing the extent of niche overlap with the two native *Vespa* species found in Europe, namely *Vespa crabro* and *Vespa orientalis*. The niches of both native hornet species exhibit partial overlap with the niche of the invasive species, as indicated by Schoener's *D* values of 0.43 for *Vespa crabro* and 0.28 for *Vespa orientalis*. Moreover, Barbet‐Massin et al. (2018), found a significant difference in the climatic niche occupied by *Vespa velutina nigrithorax* between the periods 2004–2010 and 2011–2015. Specifically, there was only a 45% overlap between these two niches. Statistical tests confirmed that while the niches were similar, they were not equivalent. The fact that the niches were not equivalent implies that during the period from 2011 to 2015, *Vespa velutina* had evolved to occupy a somewhat different climatic niche than was inhabited between 2004 and 2010. This suggests that as of 2010 and perhaps continuing to the present, the species had not yet reached a state of equilibrium with its European environment, indicating ongoing adaptation and expansion.

There is a significant knowledge gap when it comes to understanding the geographic (G‐space) and environmental (E‐space) niche overlap between hornets and honeybees, particularly in Asia. This lack of attention to niche overlap can hinder our ability to predict and effectively manage the future impacts of invasive hornets in the region. Therefore, this study aims to address critical questions within the context of hornets and honeybees in Asia. Firstly, it seeks to determine whether hornets exhibit similar and equivalent environmental niche spaces (E‐space niches) to honeybees, shedding light on potential differences in their habitat preferences. Secondly, it investigates whether hornets in Asia have reached an equilibrium geographically with honeybee species, such as *Apis cerana* and *Apis mellifera* which can help assess the extent of their ecological impact. The global distribution of western honeybee, *Apis mellifera*, encompasses a vast and varied range across Europe, Africa, and the Middle East (Han et al., 2012). While, the native range of *Apis cerana* includes most of Asia and extends from south‐eastern Asia to Russia including Japan, India, and the Middle East. It has been introduced in New Guinea, Australia, Vanuatu, and the Solomon Islands (Rojas‐Sandoval, 2022). Lastly, the study explores how the geographic space overlap (G‐space) between hornets and honeybees might change in response to climate change scenarios.

Exploring whether hornet species in their native ranges exhibit distinct E‐space niches with honeybees is crucial. If they do, it suggests that different hornet species might occupy different environmental niches in potential non‐native areas, potentially affecting native honeybee populations differently. Conversely, if these hornet species have indistinguishable E‐space niches with honeybees, they would occupy most of the areas where honeybees reside. Therefore, it is essential to determine whether hornets are in equilibrium with honeybee species like *Apis cerana* and *Apis mellifera* in Asia because, until the final stages of invasion, an invasive species has not yet reached a state of equilibrium with its new environment (Gallien et al., 2012). Understanding whether hornets can occupy all suitable environmental niches of honeybees in Asia can provide insights into their future potential impacts on native bee populations. Climate change is another significant factor to consider, as it has the potential to alter the distribution of both hornets and honeybees. Changes in temperature and precipitation patterns can influence their geographic distributions and, consequently, their G‐space overlap. Monitoring these changes and their impacts on invasive species and native ecosystems is crucial for effective management and conservation efforts. Addressing these research gaps regarding niche overlap, equilibrium dynamics, and the influence of climate change is essential for a comprehensive understanding of the interactions between hornets and honeybees in Asia and for developing strategies to mitigate potential ecological disruptions.

## 2 METHODS

### 2.1 Study area and species data

Asia is home to at least eight native honeybee species, with the greatest diversity found in tropical regions (Chantawannakul et al., 2016; Crane, 1999; Oldroyd & Nanork, 2009). For our study, we selected two widely distributed species as representatives of other *Apis* species: the native *Apis cerana* and the non‐native *Apis mellifera*. We chose these species because they are broadly representative and because other species, such as *Apis laboriosa* and *Apis dorsata*, have already been modeled under climate change scenarios in previous studies (Huang et al., 2022). Therefore, this study includes two species of honeybees as highly social species are good flagships but less‐studied groups (Junqueira et al., 2022; Pardo & Borges, 2020; Warrit et al., 2023); *Apis cerana*, *Apis mellifera*, and 12 species of hornets; *Vespa crabro*, *Vespa mandarinia* Smith, *Vespa simillima* Smith, *Vespa velutina*, *Vespa affinis*, *Vespa analis* Fabricius, *Vespa basalis* Smith, *Vespa bicolor* Smith, *Vespa ducalis* Smith, *Vespa dybowskii* André, *Vespa soror* du Buysson, *and Vespa tropica*. We obtained the presence data of these species from the Global Biodiversity Information Facility (GBIF) website (www.gbif.org).

**FIGURE 1:**
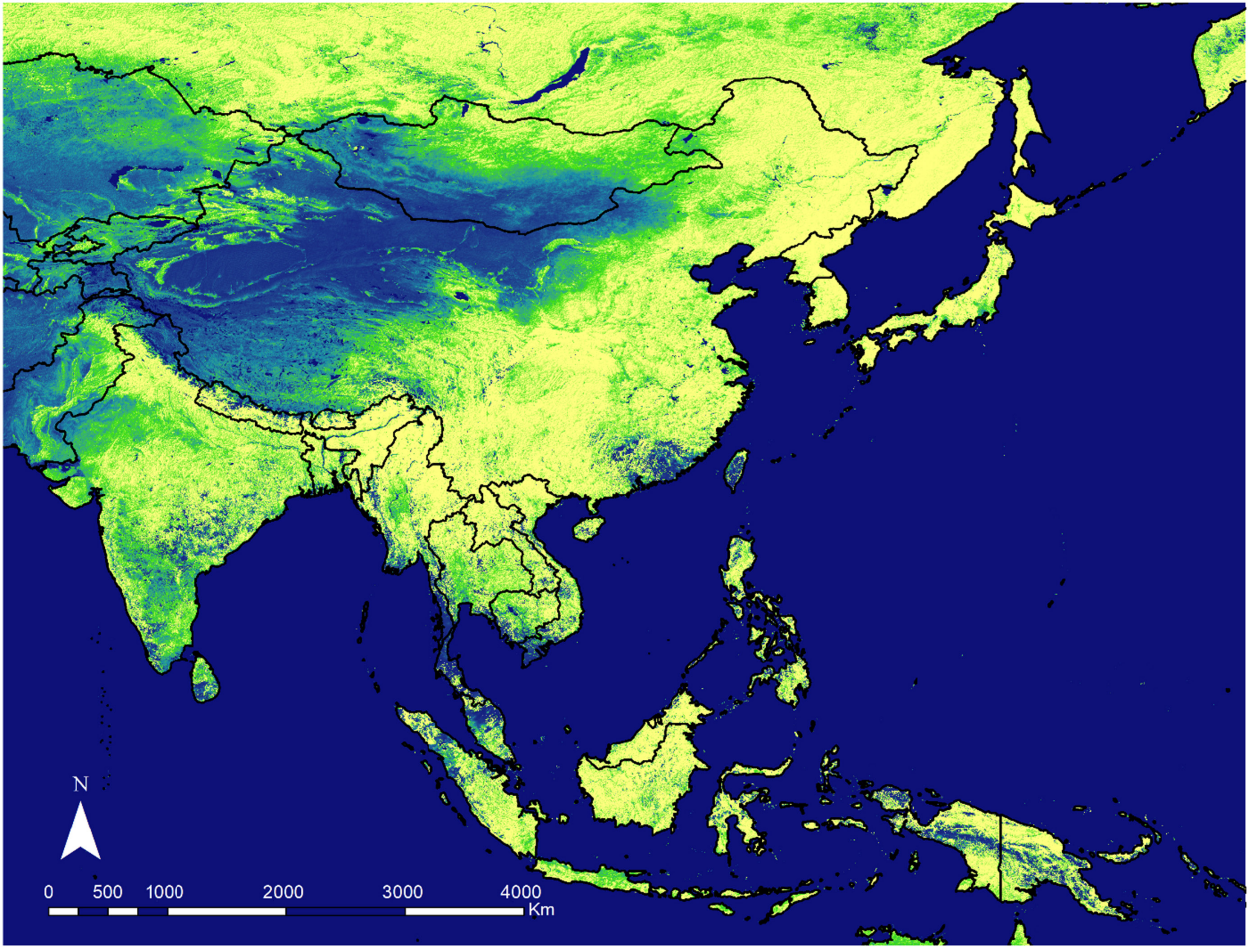
The location of the study area on the eVIRS NDVI (Normalized Difference Vegetation Index) on September 1, 2023 (EarthExplorer (usgs.gov)). Light colors (yellow) represent vegetated areas, while darker or blue areas indicate regions with low vegetation density. The deep ocean water is depicted in a deep navy blue.

In a taxonomic study, Smith‐Pardo et al. (2020) described 22 species of *Vespa* genus and their global distributions. We selected 12 hornet species for our study for two primary reasons. First, some species are not widely distributed in Asia; for example, *V. bellicosa* and *V. fervida* are found only in Indonesia, *V. luctuosa* is found only in the Philippines, and *V. orientalis* is predominantly found in North Africa and the Middle East, not in East Asia (Smith‐Pardo et al., 2020). Second, our selection was limited by the availability of occurrence data for these species from the GBIF website. To enhance data accuracy, a thorough examination of presence points was conducted to eliminate duplicate entries and records falling outside the study area boundary. Moreover, to mitigate potential biases arising from the clustering or overrepresentation of specific areas in the presence of data, spatial thinning was employed using the spThin package (Aiello‐Lammens et al., 2015). The spatial thinning distance was set at 5 km, a choice based on the scale or pixel size of the predictor layers in the model. This decision was made to ensure that the spatial thinning distance was equal to or greater than the scale to effectively reduce any spatial autocorrelation (Rodríguez‐Aguilar et al., 2023). The “rgbif” package (Chamberlain et al., 2022) was utilized to filter the records located in oceans (Figure 1).

**TABLE 1:** Model validation metrics, True Skill Statistic (TSS), and Area Under the Receiver Operating Characteristic Curve (AUC), for different taxa.

Species	AUC	TSS
*Apis cerana*	0.94	0.73
*Apis mellifera*	0.98	0.88
*Vespa bicolor*	0.98	0.91
*Vespa affins*	0.96	0.79
*Vespa analis*	0.97	0.85
*Vespa basalis*	0.96	0.83
*Vespa crabro*	0.98	0.90
*Vespa ducalis*	0.99	0.92
*Vespa dybowskii*	0.99	0.96
*Vespa mandarinia*	0.99	0.92
*Vespa simillima*	0.98	0.94
*Vespa soror*	0.97	0.87
*Vespa tropica*	0.96	0.80
*Vespa velutina*	0.97	0.82

### 2.2 Environmental data

Environmental variables, obtained from the WorldClim database (www.worldclim.org), consisted of bioclimatic layers. These bioclimatic data encompassed 11 temperature‐related variables and 8 precipitation‐related variables, all characterized by a spatial resolution of approximately 4 km. Given that these 19 variables often exhibit high levels of correlation among themselves, it is generally advised against using all of them in species distribution modeling. To address this issue, we employed the “usdm” (Naimi, 2017) package to systematically exclude highly correlated variables from the set. This was accomplished through a stepwise procedure based on the Variance Inflation Factor (VIF). Consequently, the retained variables included Mean Diurnal Range (Bio 2), Isothermality (Bio 3), Temperature Seasonality (Bio 4), Mean Temperature of Wettest Quarter (Bio 8), Mean Temperature of Driest Quarter (Bio 9), Precipitation of Wettest Month (Bio 13), Precipitation of Driest Month (Bio 14), Precipitation Seasonality (Bio 15), Precipitation of Warmest Quarter (Bio 18), Precipitation of Coldest Quarter (Bio 19).

### 2.3 Calculating E‐space niche overlap, equivalency, and similarity

To assess the extent of shared environmental niche space among hornets and honeybees, we computed ecological niche characteristics. Specifically, we calculated the niche breadth for each species, which represents the amount of ecological niche space available to the various species. Niche breadth values range from 0 to 1, where a value of 0 indicates that only one grid cell has non‐zero suitability, and a value of 1 indicates that all grid cells within the study area are equally suitable. Consequently, species with broader environmental distributions exhibit higher niche breadth values.

To extract the ecological niche space occupied by each species and quantify niche overlap, equivalence, and similarity, we employed an ordination technique. This technique applies kernel smoothers to the presence data of a species in environmental space, allowing for the selection, combination, and weighting of environmental variables (Broennimann et al., 2012). We divided the environmental space into a grid comprising 100 × 100 cells, where each cell represents a unique vector of available environmental conditions within the study area. In this study, the niche overlap between pairs of hornets and honeybees was calculated using Schoener's *D* statistic (Warren et al., 2010), directly derived from the ecological niche space. The value of *D* varies between 0, indicating no overlap in the environmental space between the two species, and 1, signifying that the two species share the same environmental space.

We employed the niche equivalence test to evaluate whether the ecological niches of pairs of hornets and honeybees are significantly distinct from each other (i.e., whether the two niche spaces are interchangeable). To conduct the niche equivalence test, we compared the niche overlap values (*D*) of pairs of hornet and honeybee species to a null distribution consisting of 100 overlap values. We inferred equivalence of ecological niches when the niche overlap value of the species being compared was not significantly (*p* ≤ .05) lower or higher than the overlap values from the null distribution.

The test for niche equivalence is considered conservative because it solely evaluates whether two species have identical niche spaces based on their precise locations, without taking into account the surrounding space. Therefore, we also conducted a niche similarity test, which assesses whether the ecological niches of each pair of species are more dissimilar than expected by chance. This test accounts for variations in the surrounding environmental conditions within the geographic areas where the two species co‐occur (Warren et al., 2010). A significant difference detected by the niche similarity test would not only imply differences in the environmental niche spaces occupied by the two species but also that these distinctions are not attributable to the geographic availability of environmental conditions.

Additionally, in the case of E‐space analysis, the niche overlap can be disentangled into three categories: unfilling, stability, and expansion (Di Cola et al., 2017). Unfilling typically represents the fraction of the niche of prey (honeybees) that does not overlap with the niche of predators (hornets). Conversely, expansion denotes the portion of the niche of predator that lacks overlap with the niche of prey. Niche stability is the proportion of the niche of prey overlapping with the niche of predator (Guisan et al., 2014). All niche overlap analyses were conducted using the “ecospat” package (Di Cola et al., 2017) in R software.

### 2.4 Climate change and G‐space niche overlap

In this study, we employed the SDM package (Naimi et al., 2016) within the R 4.2.3 software environment to model the distribution of each species under investigation. Specifically, we utilized the MaxEnt model to generate habitat suitability maps, drawing upon both presence data and climatic information. The MaxEnt model is a widely accepted tool frequently used in diverse studies to model species distribution and estimate the potential impacts of climate change, particularly in the context of insects (Aguirre‐Gutiérrez et al., 2013; Filazzola et al., 2020; Huang et al., 2022; Martínez‐López et al., 2021; Miličić et al., 2018; Rahimi et al., 2021; SBARAGLIA, 2022; Tabor & Koch, 2021). The performance of the MaxEnt model can be similar to that of the ensemble approach. Additionally, the MaxEnt method offered reduced computational time and greater simplicity (Kaky et al., 2020). To enhance our modeling efforts, we generated 10,000 pseudo‐absence points to complement the presence points when applying the MaxEnt model. Furthermore, we incorporated a climate change scenario, namely SSP585 (the shared socio‐economic pathway), to project the future distribution of species in 2070. SSP585 represents an extreme climate change scenario characterized by a tripling of CO2 emissions by 2075 and a 4.4‐degree temperature increase by 2070 (O'Neill et al., 2016).

The habitat suitability maps generated in this study have values ranging from 0 to 1, with 1 representing the highest level of suitability. To assess the potential impacts of climate change on the future G‐Space niche overlap between hornets and honeybees, we also utilized Schoener's *D* statistic. This index was employed to quantify their overlap in both current and projected future scenarios, allowing us to examine how climate change might alter the degree of overlap between each pair of hornet and honeybee species. We conducted mutual comparisons among 91 pairs of 14 species (2 honeybee and 12 hornet species), calculating the shorter D statistic for all pairs under both current and future scenarios.

To present how climate change may affect the future distribution of the studied species, we selected four representative species: *A. mellifera*, *A. cerana*, *V. mandarinia*, and *V. velutina*. We classified their continuous distribution maps into three categories: increase, decrease, and no change. To achieve this, we subtracted the future maps of these species from their current ones. The result is a map showing pixel values that are positive (indicating an increase), negative (indicating a decrease), or zero (indicating no change). Finally, we classified all positive values as the increase category, negative values as the decrease category, and zeros as the no change category.

### 2.5 Model assessment

To assess the performance of the different models' results, we employed two key statistical metrics: The True Skill Statistic (TSS) and the Area Under the Receiver Operating Characteristic Curve (AUC). AUC values typically fall within the range of 0.7 to 0.9, indicating acceptable model performance. Values exceeding 0.9 are indicative of excellent results, signifying that the model has provided highly accurate predictions. TSS values, on the other hand, lie between −1 and +1. A TSS of 1 signifies a perfect and entirely accurate prediction, while values ranging from 0.4 to 0.6 suggest moderate predictive performance. In our evaluation process, we conducted 10 runs of subsampling replications. During each run, 70% of the presence data were utilized for training the model, while the remaining 30% were reserved for testing the model's predictions.

## 3 RESULTS

### 3.1 Model assessment

Table 1 presents the model validation metrics results for bees and hornets, utilizing two statistics, Area Under the Receiver Operating Characteristic Curve (AUC) and True Skill Statistic (TSS). These metrics were evaluated for all the studied species; the table reports the average AUC and TSS values for each species. The results indicate that the AUC values for all species range between 0.96 and 0.99, suggesting excellent model performance. Additionally, based on the TSS test.

### 3.2 E‐space niche overlap

**FIGURE 2:**
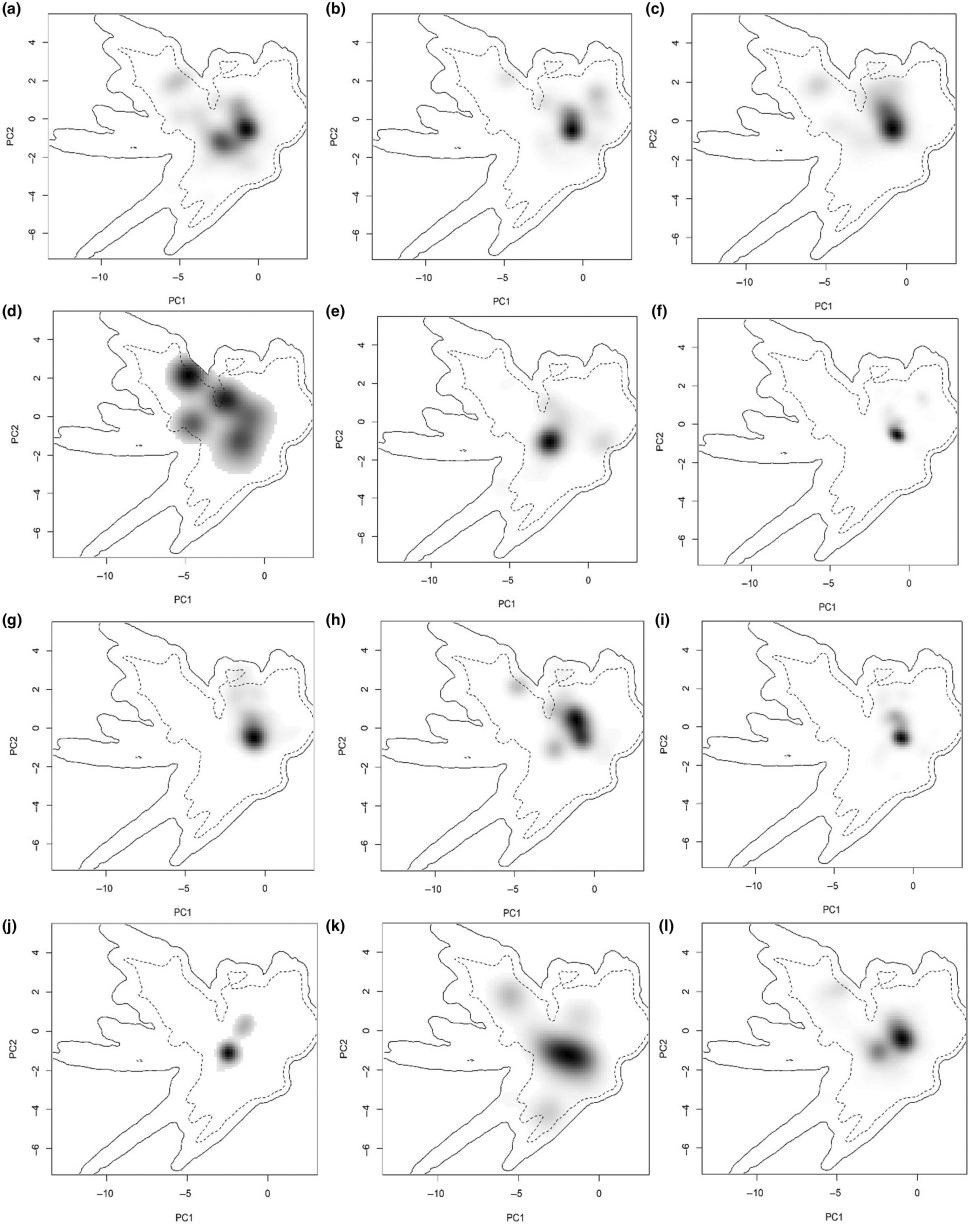
Niche breadth of 12 analyzed hornets and honeybees in environmental space produced by the principal component analysis method (PCA‐ent). The grey‐to‐black shading represents the grid cell density of the species occurrences (black being the highest density). The first dashed line represents 50% of the available environment and the solid line represents 100%. (a) *A. cerana*, (b) *A. mellifera*, (c) *V. analis*, (d) *V. basalis*, (e) *V. bicolor*, (f) *V. velutina*, (g) *V. dybowskii*, (h) *V. ducalis*, (i) *V. mandarinia*, (j) *V. soror*, (k) *V. tropica*, (l) *V. velutina*.

Figure 2 illustrates the E‐space niche of the 12 analyzed hornets and two honeybees within the environmental space generated by the principal component analysis (PCA‐ent) method. The PCA‐ent results depict the niche of each species in the two primary axes relative to the environmental conditions across the entire study region. The shading, ranging from gray to black, represents the grid cell density of species occurrences, with black indicating the highest density. The first dashed line signifies 50% of the available environment, while the solid line denotes 100%.

Indeed, the analysis of Figure 4 indicates that certain species, including *Apis cerana*, *Apis mellifera*, *Vespa crabro*, *Vespa velutina*, *Vespa affinis*, *Vespa analis*, *Vespa basalis*, and *Vespa tropica*, share regions in the PCA plots for the highest density of occurrences. This suggests that these species exhibit similarities in their preferred environmental niches, leading to the clustering of their occurrence points in the same portions of the environmental space. However, a comprehensive examination of all the plots reveals distinctions in both niche breadth and the locations of density peaks among these species. While some species may share certain environmental preferences and, as a result, exhibit overlapping occurrences in specific areas, the variation in their niche breadth and density locations emphasizes that each species has its unique ecological niche.

### 3.3 Niche equivalency and similarity

In every conceivable pairwise comparison among hornets and honeybees, we rejected the null hypothesis of the niche equivalency test. Conversely, when assessing niche similarity, we found that the null hypothesis was upheld for some pairs (Table 2). Most of the pairs (see Data S1 and S2) of hornets and honeybees exhibited niche similarities that did not exceed what would be expected by chance. Therefore, we directed further attention to the species pairs with significant niche similarity tests (Table 2). According to Table 2, *Apis cerana* and *Apis mellifera* notably share remarkably similar and equivalent climatic niches, and this similarity is statistically significant (*p*‐value < .05). Furthermore, when examining niche expansion, unfilling, and stability, it becomes evident that the disparities between their climatic niches are minimal. *Apis cerana* exhibits a noteworthy non‐random similarity with three hornet species, with the highest degree of similarity observed with *Vespa velutina* (Schoener's *D* = 0.74). The outcomes of the niche expansion, unfilling, and stability assessments reinforce the notion that *Vespa velutina* and *Apis cerana* possess climatic niches that are akin and virtually fully occupied, leaving little unexplored environmental niche space within *Apis cerana's* niche for *Vespa velutina* to occupy. A parallel conclusion can be drawn for other hornets like *Vespa ducalis* and *Vespa basalis*. Furthermore, *Apis mellifera* also shares a climatic niche that is akin and commensurate with hornets such as *Vespa velutina* and *Vespa ducalis*.

**TABLE 2:** Niche comparisons for the hornets and honeybees. All of the comparisons between the hornets and honeybees highlight the non‐equivalency of their ecological niches.

Pairs	Overlap		Niche similarity			Niche equivalency
	*D*	Expansion	Stability	Unfilling	*p*‐value	*p*‐value
*A. cerana* vs. *V. ducalis*	0.62	0.02	0.98	0.23	.02	.18
*A. cerana* vs. *V. velutina*	0.74	0.00	0.99	0.03	.02	.27
*A. cerana* vs. *V. basalis*	0.59	0.02	0.98	0.07	.02	.97
*A. cerana* vs. *A. mellifera*	0.64	0.09	0.91	0.07	.04	1
*A. mellifera* vs. *V. ducalis*	0.63	0.00	0.99	0.19	.01	1
*A. mellifera* vs. *V. velutina*	0.55	0.04	0.96	0.14	.03	1
*V. velutina* vs. *V. ducalis*	0.75	0.04	0.96	0.12	.01	.09
*V. velutina* vs. *V. basalis*	0.65	0.10	0.90	0.03	.03	.77
*V. basalis* vs. *V. ducalis*	0.63	0.04	0.96	0.32	.04	1
*V. crabro* vs. *V. mandarinia*	0.52	0.07	0.93	0.09	.04	.92

**FIGURE 3:**
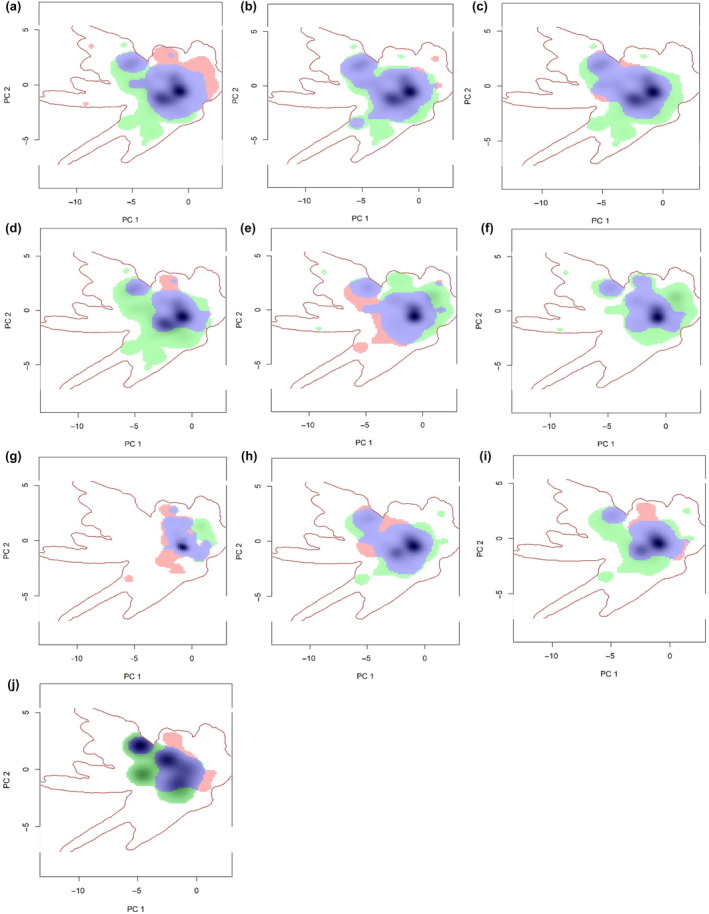
Climate niche surfaces overlaid between hornets and honeybees for 10 pairs. The pair of surfaces for each species was created on one of three background extents that gave the best environmental niche model transferability. The first two axes from the underlying principal components analysis are shown on the x‐axis (PC1) and y‐axis (PC2). Within each plot, the green area indicates niche unfilling, purple indicates niche stability and red indicates niche expansion. The solid lines indicate 100% of available climates for each respective background (a) *A. cerana* vs *A. mellifera*, (b) *A. cerana* vs *V. velutina*, (c) *A.ceranavs* *V. basalis* (d) *A. ceranavs* *V. ducalis*, (e) *A. mellifera* vs *V. velutina*, (f) *A. mellifera* vs *V. ducalis*, (g) *v. crabro* vs *V. mandarinia*, (h) *V. velutina* vs *V. basalis*, (i) *V. veluytina* vs *V. ducalis*, (j) *V. basalis* vs *V. ducalis*.

In alignment with the findings presented in Table 2, we have visualized the patterns of niche overlap between species. Specifically, Figure 3 illustrates the climate niche surfaces that overlap between hornets and honeybees for a total of 10 species‐pairs. Each pair of niche surfaces was generated within one of three background extents that yielded the most effective transferability for environmental niche modeling. The x‐axis (PC1) and y‐axis (PC2) of each plot represent the first two principal components derived from the underlying principal components analysis. In these plots, the green area signifies niche unfilling, the purple area represents niche stability, and the red area indicates niche expansion. Additionally, the solid lines demarcate 100% of the available climates for each respective background, while the dashed lines correspond to 75% of the available climates.

### 3.4 Potential effects of climate change

**FIGURE 4:**
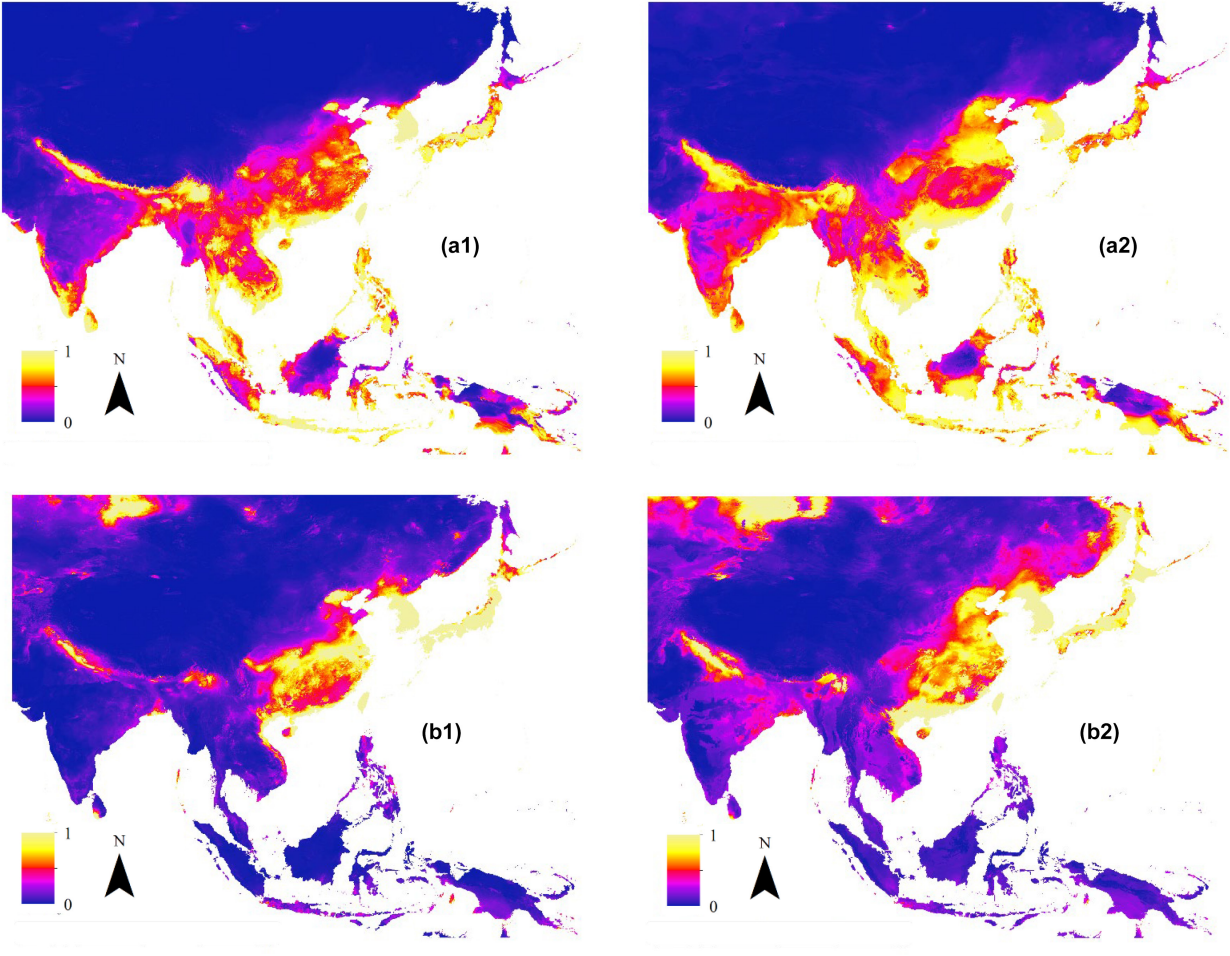
Habitat suitability maps in current and future scenarios. Number 1 shows current and number 2 shows future potential distributions. (a1/a2) *A. cerana*, (b1/b2) *A. mellifera*.

Figure 4 displays habitat suitability maps for several hornets and honeybees, illustrating current and potential future distributions. The maps represent *A. cerana* (a1/a2), *A. mellifera* (b1/b2), *V. velutina* (c1/c2), *V. mandarinia* (d1/d2), *V. affinis* (e1/e2), and *V. basalis* (f1/f2). According to this visual analysis, it suggests that by 2070, under the worst climate change scenario, the range of *A. cerana* and *A. mellifera* is expected to expand notably, especially in the northern regions of India. Similarly, *V. velutina* shows a comparable trend of potential distribution towards higher latitudes.

**FIGURE 5:**
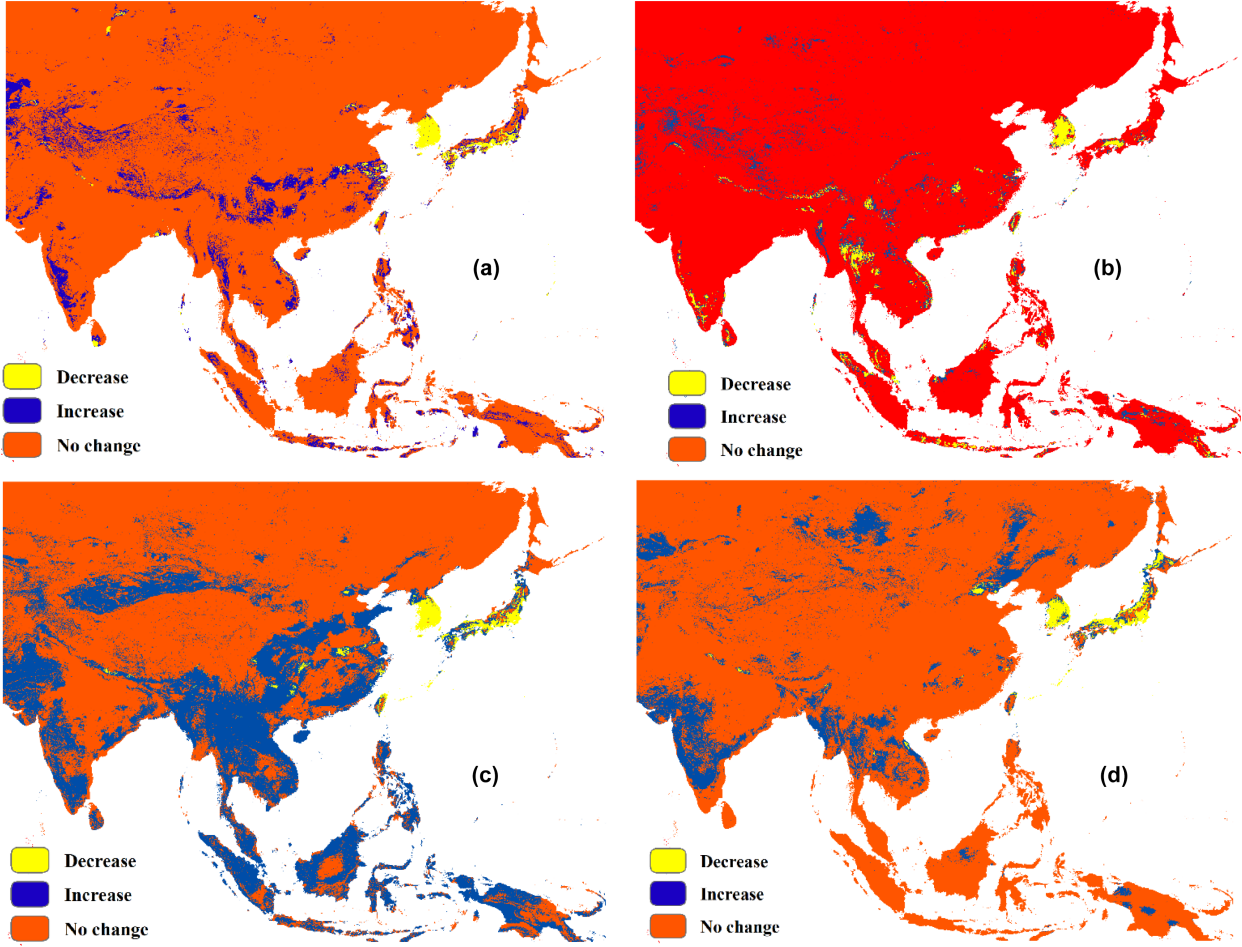
Potential changes in future distribution of (a) *A. mellifera*, (b) *A. cerena*, (c) *V. mandarinia*, and (d) *V. velutina*. Areas exhibiting a potential increase are depicted in blue, while those showing a potential decrease are highlighted in yellow. Regions where no change is expected are indicated in red.

We also analyzed future distribution maps for four species and classified the changes as positive or negative in distribution cells. Figure 5 illustrates the potential changes in the distribution of (a) *A. mellifera*, (b) *A. cerana*, (c) *V. mandarinia*, and (d) *V. velutina*. According to the figure, honeybees are expected to experience an increase in the northern parts of India and western parts of China, with some decreases in South Korea and Japan. On the other hand, *V. mandarinia* shows a significant future distribution across Asia, with some decreases in habitat suitability in South Korea and Japan. Similar results can be observed for *V. velutina*, although the magnitude of changes is less pronounced compared to *V. mandarinia*.

**FIGURE 6:**
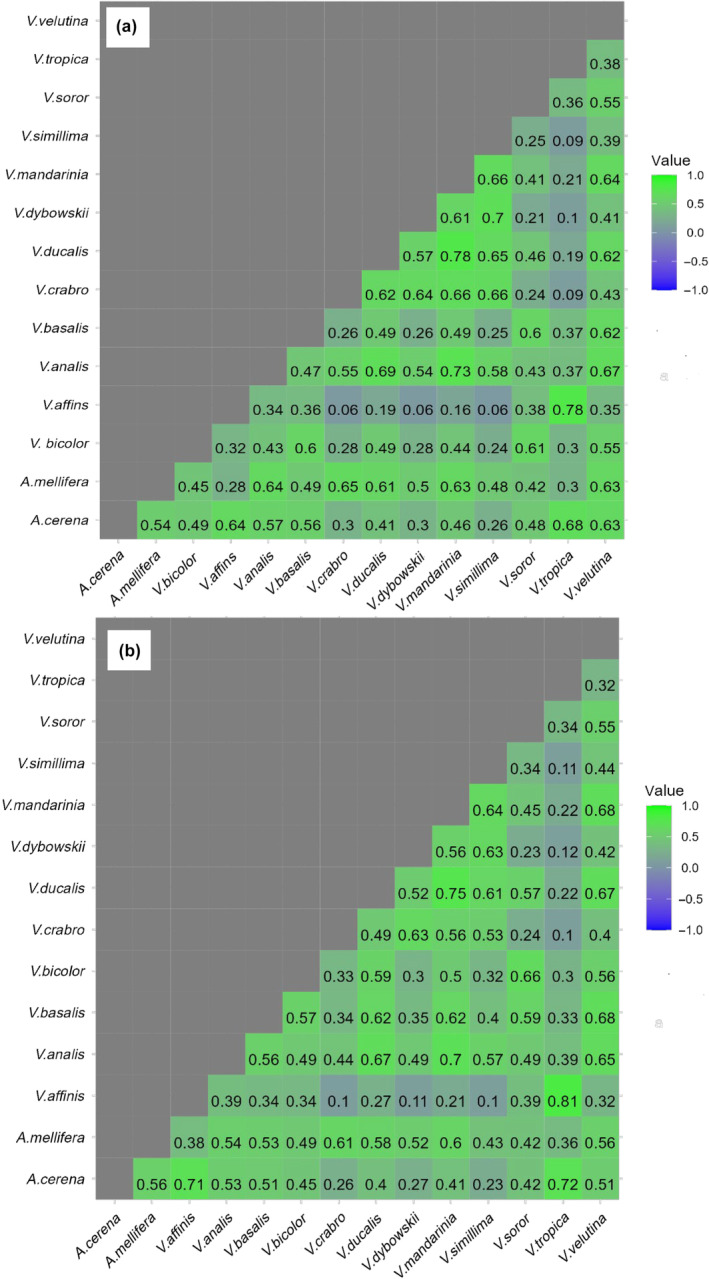
Schoener's *D* statistic for 91 pairs of hornets and honeybees in current (a) and future (b) scenarios.

Figure 6 presents the geographic niche overlap between all 91 pairs of species in the current scenario (a) and future scenarios (b). Our primary focus is on the overlap between hornets and honeybees. According to this figure, the geographic overlap between *Vespa velutina* and both honeybees, *Apis cerana* and *Apis mellifera*, is expected to decrease slightly. Specifically, the overlap is projected to decrease from 0.63 in the current scenario to 0.51 for *Apis cerana* and 0.56 for *Apis mellifera* in the future scenario. *Vespa velutina* is anticipated to undergo a decrease in overlap with both honeybees, suggesting a potential shift in their distribution patterns. *Vespa tropica* is anticipated to experience an increase in overlap with both honeybees. Conversely, *Vespa soror* shows a decrease in overlap, albeit affecting only *Apis cerana*, signaling a potential reduction in their cohabitation. *Vespa simillima* is expected to undergo a decrease in overlap with both honeybees, implying a shift in their distribution patterns. While *Vespa mandarinia* may see a slight decrease in overlap, *Vespa dybowskii* exhibits a minor increase with *Apis mellifera* and a slight decrease with *Apis cerana*. *Vespa ducalis* is projected to have a slight decrease in overlap with both honeybees. For *Vespa crabro*, there will likely be an increase in overlap with *Apis cerana* and a minor decrease with *Apis mellifera*. *Vespa bicolor* is poised for a slight increase in overlap with *Apis mellifera* but a minor decrease with *Apis cerana*. *Vespa basalis* shows an increase in overlap with *Apis mellifera* but a decrease with *Apis cerana*. Lastly, both *Vespa analis* and *Vespa affinis* are expected to experience an increase in overlap with both honeybees.

## 4 DISCUSSION

### 4.1 E‐space niche overlap

Our results demonstrated that the climatic niches of hornets and honeybees can be treated as interchangeable. The rejection of the null hypothesis in our assessment of niche equivalency emphasizes that the ecological niches of all species pairs are equivalent. Inconsistent with other studies (Aguirre‐Gutiérrez et al., 2015), this underscores the legitimacy of inferring the niche characteristics of one hornet based on another, when these species are considered closely related. Niche similarity results reveal that some hornets share more commonalities with honeybees in their environmental niche spaces than would be expected by chance. This suggests a strong potential interaction between these species, indicating they have similar climate preferences despite being unrelated taxa.

In the context of E‐space niche analysis, some studies have relied solely on extrapolation, predicting potential invasion locations by considering the native ranges of hornets (Bessa et al., 2016; Herrera et al., 2023; Moo‐Llanes, 2021; Nuñez‐Penichet et al., 2021). Others have conducted niche overlap analyses that compare native and non‐native ranges of hornets to determine if these species exhibit niche conservatism. For example, Barbet‐Massin et al. (2018) tried to determine whether *Vespa velutina* has reached equilibrium in its invaded range in Europe. To do this, they compared the climatic niche that the species occupied during the initial phase of its invasion (from 2004 to 2010) with the niche it currently occupies based on more recent data from 2011 to 2015. Their analysis revealed a significant difference in the climatic niche occupied by *Vespa velutina* between the periods 2004–2010 and 2011–2015. Statistical tests confirmed that while the niches were similar, they were not equivalent. Nie et al. (2024) Utilized land‐use, climate, and topographical data, they projected the range dynamics of *V. velutina* and *A. mellifera* in Europe, along with their future range overlap. They found that the dominant influence of climatic factors on the potential ranges of hornets, surpassing the effects of land use and topography, and future expansions of the hornet's range are forecasted in the UK and France. However, our findings indicated that *Vespa velutina* is in climatic equilibrium with honeybees, and the assessments of niche stability, unfilling, and expansion between them suggested that there were limited opportunities within the climatic niche of honeybees for *Vespa velutina* to occupy.

Other studies have evaluated E‐space niche overlap between native and non‐native ranges of individual species (e.g., *V. velutina*) or analyzed the niches between different hornet species. For example, Lioy et al. (2023) assessed the extent of niche overlap between *V. velutina* and two native hornet species that inhabit Europe, namely *Vespa crabro* and *V. orientalis*. Their results indicate that the niches of both native species exhibit partial overlap with the niche of the invasive species, as evidenced by Schoener's *D* values of 0.43 with *V. crabro* and 0.28 with *V. orientalis*. However, these findings also reveal distinctions among the niches. *V. crabro* seems better adapted to cold and dry environmental conditions compared to the invasive *V. velutina*, while *V. orientalis* appears to be better adapted to arid climates. These distinctions may confer a competitive advantage to the native species in regions with relatively low environmental suitability for *V. velutina*, particularly if the invasive species continues its spread and reaches all areas predicted to be suitable in Europe and the Mediterranean basin. Nuñez‐Penichet et al. (2021) investigated the impact of *Vespa velutina* abundance on the European hornet *V. crabro* in northern Italy where *V. velutina* had recently become established. Additionally, they assessed the effects of *V*, *velutina* on *V. crabro*, *Vespula vulgaris*, and *Vespula germanica* by comparing between the invaded area and an uninvaded region. They discovered that a positive correlation between the two hornet species at low abundances. However, when *Vespa velutina* populations reached high levels, the two species ceased to covary. Notably, the distribution patterns of *V. crabro*, *V. vulgaris*, and *V. germanica* displayed substantial overlap between the invaded and uninvaded areas. Overall, their findings led to the conclusion that native Vespidae species have likely managed to avoid or mitigate competition pressure. Consequently, the presence of *Vespa velutina* has not resulted in an evident displacement of *V. crabro* or the *Vespula* species. This offers reassurance regarding the conservation status of native European Vespidae in the wake of *Vespa velutina's* invasion.

In invaded areas, the lifecycle of *V. velutina* typically begins with the emergence of the foundress from hibernation, nest construction, and egg‐laying, occurring from January to April (Monceau et al., 2014). Worker hornets emerge in May, and throughout the summer, the colony and nest grow in size. By late summer, when worker numbers peak and the reproductive brood requires feeding, hornet predation intensifies (Rome et al., 2011). Secondary nest construction typically occurs around August. During autumn, mating activities involving gynes and males predominate, followed by dispersal. The cycle concludes with gynes overwintering (Lima, 2020). The biological and ecological responses of hornets to temperature changes are crucial, and further studies in this area are essential to gain a deeper understanding of its behavior and potential impacts. For example, Keeling et al. (2017) evaluated the likelihood of *V. velutina* invading Great Britain and emphasized the uncertainty regarding its behavior, particularly its response to the colder climate in comparison to France. Spiewok and Schmolz (2006) also show that ambient temperature and illuminance influence the flight performance of hornets, with drones being negatively correlated with ambient temperature.

This section concludes that hornets and honeybees share a comparable climate niche, suggesting that if non‐native hornets (like *Vespa velutina*) enter the geographic area of honeybees, they may entirely occupy and impact the entire territory or population of honeybees. Nevertheless, other factors like competition and biotic interactions could constrain the spread of hornets.

### 4.2 Climate change and G‐space niche overlap

Our findings in this section reveal that honeybees and hornets already share a moderate to high geographic niche overlap, which is reasonable since not all hornet species occupy the same geographic space as honeybees in Asia. Consequently, there exist certain geographic regions where the two species of honeybees coexist but do not overlap with hornet distributions. This suggests that hornets are not in geographic equilibrium with honeybees, signifying variations in their spatial distribution patterns. Under climate change modeling, species like *V. crabro*, *V. soror*, *V. velutina*, *V. analis*, *V. basalis*, *V. ducalis*, *V. dybowskii*, *V. mandarinia*, *V. simillima*, and *V. bicolor* are projected to reduce their overlap with *A. cerana*. On the other hand, *V. affinis and V. tropica* might increase their overlap with *A. cerana*. Concerning *A. mellifera*, *V. velutina*, *V. simillima*, *V. ducalis*, *V. crabro*, *V. analis*, and *V. mandarinia* are anticipated to decrease their overlap. Meanwhile, *V. tropica*, *V. dybowskii*, *V. basalis*, *V. affinis*, and *V. bicolor* may experience an increase in their overlap with this species. These findings suggest that climate change may have varying impacts on different species within the hornet and honeybee groups, potentially affecting their interactions and ecological dynamics. While the overlap values of Schoener's *D* statistic between hornets and honeybees are anticipated to undergo slight changes, comprehending these shifts is crucial for evaluating the implications of climate change on these significant pollinators and their respective ecosystems.

No previous study has utilized Schoener's *D* statistic to evaluate the current and future overlap between hornets and honeybees. Consequently, making a direct comparison between our findings and those of other studies is challenging. Nevertheless, some studies have focused on species distribution modeling of hornets, such as *Vespa velutina*, to forecast the potential invasion of this species into other regions globally. For instance, Villemant et al. (2011) employed several ensemble models to predict the invasion risk *Vespa velutina* across Europe and other continents. They found that the most suitable areas in Europe to be predominantly along the Atlantic coast, the Mediterranean coast, and the southern shores of the Black and Caspian Seas. Barbet‐Massin et al. (2013) also aimed to assess the potential impact of climate change on invasion risk. They extended the models to project future scenarios up to 2100. They found that climate change is likely to elevate invasion risk in the United States, except for areas along the Eastern coast. Nonetheless, our results indicated a significant decrease in invasion risk for certain hornet species in Asia. However, it's important to note that adaptability to climatic conditions is just one facet influencing the invasion risk of hornets. Other crucial factors include interspecific hierarchies, competition for prey resources, temporal overlap in seasonal phenology, competition for nesting sites, aggressiveness, and body size (Kwon & Choi, 2020; Lioy et al., 2022, 2023).

## 5 CONCLUSION

Our investigation has shed light on the complex interplay between honeybees and hornets in terms of geographic and climatic niche overlap and the potential impacts of climate change. We showed that the climatic niches of these two groups of species (hornets and honeybees) can be considered interchangeable in Asia. However, this climatic similarity in their niches does not extend to their geographical distribution. In other words, while hornets can occupy most of the areas where honeybees are residing in Asia, they are limited by geographical barriers. This implies that if hornets were to gain access to honeybee habitats that are currently beyond their range, they have the potential to occupy those habitats. This suggests a certain level of ecological plasticity in hornet species, enabling them to exploit a broader range of habitats than they currently inhabit. Furthermore, it is important to consider that climate change or other environmental alterations could create new opportunities for species like hornets to expand their ranges. As environmental conditions shift, some habitats that were once inaccessible to hornets may become more suitable for them, potentially leading to changes in their geographical distribution and interactions with honeybees. These findings contribute to our understanding of the intricate ecological dynamics between hornets and honeybees, highlighting the unique aspects of their niche interactions and the potential implications for their coexistence in changing environments.

## AUTHOR CONTRIBUTIONS

**Ehsan Rahimi:** Conceptualization (equal); formal analysis (equal); methodology (equal); software (equal); writing – original draft (equal). **Chuleui Jung:** Project administration (equal); resources (equal); supervision (equal); writing – review and editing (equal).

## FUNDING INFORMATION

This research was funded by RDA Korea, grant number RS‐2023‐00232847, and National Research Foundation of Korea (National Research Foundation of Korea (NRF‐2018R1A6A1A03024862)).

## CONFLICT OF INTEREST STATEMENT

On behalf of all authors, the corresponding author states that there is no conflict of interest.

## CODE AVAILABILITY STATEMENT

R codes are available on request from the authors only based on logical requests from Ehsan Rahimi (ehsanrahimi666@gmil.com).

## Supporting information


Data S1.

Data S2.

## Data Availability

Two Excel data of similarity and equivalency tests of 91 pairs of hornets and honeybees are available at https://github.com/ehsanrahimi666/G‐Space‐versus‐E‐Space.
